# (GT)_n_ Repeat Polymorphism in Heme Oxygenase-1 (HO-1) Correlates with Clinical Outcome after Myeloablative or Nonmyeloablative Allogeneic Hematopoietic Cell Transplantation

**DOI:** 10.1371/journal.pone.0168210

**Published:** 2016-12-20

**Authors:** Tania Køllgaard, Brian Kornblit, Jesper Petersen, Tobias Wirenfeldt Klausen, Bo Kok Mortensen, Peter Brændstrup, Henrik Sengeløv, Estrid Høgdall, Klaus Müller, Lars Vindeløv, Mads Hald Andersen, Per thor Straten

**Affiliations:** 1 Center for Cancer Immune Therapy (CCIT), Department of Hematology, 54P4, Copenhagen University Hospital, Herlev, Denmark; 2 Allogeneic Hematopoietic Cell Transplantation Laboratory, Department of Hematology, Rigshospitalet, Copenhagen, Denmark; 3 The Bone Marrow Transplantation Unit, Department of Hematology, Rigshospitalet, Copenhagen, Denmark; 4 Department of Pathology, Molecular Unit, Copenhagen University Hospital, Herlev, Denmark; 5 Department of Pediatrics and Adolescent Medicine and Institute of Inflammation Research, Rigshospitalet, Copenhagen, Denmark; University of Kentucky, UNITED STATES

## Abstract

Allogeneic hematopoietic cell transplantation (HCT) is a treatment for various hematologic diseases where efficacy of treatment is in part based on the graft versus tumour (GVT) activity of cells in the transplant. The cytoprotective enzyme heme oxygenase-1 (HO-1) is a rate-limiting enzyme in heme degradation and it has been shown to exert anti-inflammatory functions. In humans a (GT)_n_ repeat polymorphism regulates the expression of HO-1. We conducted fragment length analyses of the (GT)_n_ repeat in the promotor region of the gene for HO-1 in DNA from donors and recipients receiving allogeneic myeloablative- (MA) (n = 110) or nonmyeloablative- (NMA-) (n = 250) HCT. Subsequently, we compared the length of the (GT)_n_ repeat with clinical outcome after HCT. We demonstrated that transplants from a HO-1^high^ donor after MA-conditioning (n = 13) is associated with higher relapse incidence at 3 years (p = 0.01, n = 110). In the NMA-conditioning setting transplantation of HO-1^low^ donor cells into HO-1^low^ recipients correlated significantly with decreased relapse related mortality (RRM) and longer progression free survival (PFS) (p = 0.03 and p = 0.008, respectively). Overall, our findings suggest that HO-1 may play a role for the induction of GVT effect after allogeneic HCT.

## Introduction

Allogeneic hematopoietic cell transplantation (HCT) is an effective therapeutic option for patients with various hematologic malignancies. Current challenges include reducing graft-versus-host disease (GVHD) while maintaining or augmenting the beneficial graft-versus-tumour effect (GVT) [1]. Donor-derived type 1 T cells are known to be major players in GVT and GVHD which involve killing of tumour cells as well as healthy cells and tissues, respectively [1].

Heme oxygenase-1 (HO-1) is a rate limiting stress-responsive enzyme that degrades heme into biliverdin, carbon monoxide and free iron [2]. It is induced by various oxidative agents and exerts anti-inflammatory and cytoprotective effects hereby controlling inflammation and inducing tolerance [3]. To this end, dendritic cells expressing HO-1 retain an immature phenotype which promote differentiation of Tregs and inhibit effector T-cell responses [3]. Indeed, it could be speculated that the immune suppressive functions of HO-1 may influence the immune mechanisms underlying the effects of allogeneic HCT. It has been demonstrated, that a (GT)_n_ dinucleotide repeat in the promoter region of the HO-1 encoding gene, *HMOX1*, regulates the expression of HO-1 [4]. Shorter (GT)_n_ repeats leads to increased transcriptional activity and, thus, a higher expression of HO-1 upon oxidative stimuli [5].

The purpose of our study was to examine a possible association of the association between the HO-1 (GT)_n_ repeat polymorphism and clinical outcomes after both myeloablative (MA) or nonmyeloablative (NMA) allogeneic HCT.

## Methods

### Patients and controls

The study population consisted of patients with malignant hematologic diseases that received related or unrelated donor HCT after MA conditioning (n = 110) or NMA conditioning (n = 250) (Table 1) between March 2000 and July 2007 at the bone marrow transplantation unit at Rigshospitalet, Copenhagen, Denmark. Transplant regimens and supportive care have been described elsewhere [6]. Acute GVHD and chronic GVHD were graded according to standard criteria [7]. Samples from related and unrelated donors were obtained from leukapheresis products whereas peripheral blood samples were obtained from healthy donors. DNA was extracted as previously reported using the Promega Maxwell 16 blood DNA kit (Promega Corporation, Madison, WI) [8]. Written informed consent was obtained from all recipients and donors and the study and consent procedure was approved by the Scientific Ethics Committee for The Capital Region of Denmark. Our healthy cohort included DNA from 74 individuals from the Danish general population [9].

**Table 1:** Patient demographics.

PATIENTS	Myeloablative cohort(n = 110)	Nonmyeloablative cohort(n = 250)
**Patient age, median years (range)**	40 (16–57)	56 (19–74)
**Sex of patient**		
M	66 (60%)	157 (63%)
F	44 (40%)	93 (37%)
**Disease**		
AML	44 (40%)	77 (31%)
ALL	32 (29%)	1 (0%)
MDS (de novo)	16 (15%)	40 (16%
MDS (therapy related)	0 (0%)	8 (3%
CML	15 (14%)	5 (2%)
NHL	3 (3%	46 (18%
MM	0 (0%)	12 (5%)
HD	0 (0%)	15 (6%
CLL	0 (0%)	46 (18%)
**Conditioning regimen**		
TBI (12 GY) + Cyclophosphamide	61 (56%)	-
TBI (12 GY) + Etoposide	39 (35%	-
Cyclophosphamide (+/- Busulfan)	9 (8%)	-
Fludarabine, Thiotepa and Melphalan	1 (1%)	-
TBI (2 GY) + Fludarabin	-	221(89%)
TBI (2 GY)	-	3 (1%)
TBI (3 GY) + Fludarabin	-	1 (0%)
TBI (4 GY) + Fludarabin	-	25 (10%)
**Patient HO-1 Genotype**	-	
S/S	14 (13%)	26 (10%
S/L	41 (37%)	125 (50%)
L/L	34 (31%)	99 (40%)
Missing	21 (19%)	0 (0%)
P for Hardy-Weinberg equilibrium	0.97	0.34
**Karnofsky score**		
>80	92 (84%)	191 (76%)
≤80	12 (11%)	27 (11%)
Not available	6 (5%)	32 (12%)
**Relapse risk (Kahl Score)**		
Low risk	-	62 (25%)
Standard risk	-	140 (56%)
High risk	-	48 (19%)
**Disease stage (EBMT score)**		
Early	56 (51%)	-
Intermediate	44 (40%)	-
Late	10 (9%)	-
**Overall Survival, median days**	1701	2482
**Follow-up time, median days (range)**	1585 (660–3076)	1119 (272–3229)
**DONORS**		
**Donor age, median years (range)**	**38 (20–59)**	**42 (19–69)**
**Sex of donor**		
M	73 (66%)	144 (58%)
F	37 (34%)	106 (42%)
**Donor HO-1 Genotype**		
S/S	13 (12%)	35 (14%)
S/L	40 (36%)	114 (46%)
L/L	57 (52%)	101 (40%)
P for Hardy-Weinberg equilibrium	0.37	0.95
**PATIENT/DONOR**		
**HLA match**		
10/10	95 (86%)	231 (92%)
9/10	15 (14%)	19 (8%)
**Type of donor**		
Siblings	34 (31%)	113 (45%)
Other relative	2 (2%)	1(0%)
Unrelated	74 (67%)	136 (55%)
**CMV status**		
Neg/neg	26 (24%)	65 (26%)
Other	81 (74%)	174 (70%)
Missing	3 (3%)	11 (4%)

Values are number of cases with percents in parenthesis, unless otherwise specified. TBI indicates total body irradiation,

### Fragment length analyses of the (GT)_n_ repeat in the promoter region of the HMOX1 gene

To determine the number of (GT)_n_ repeats in the promoter region, polymerase chain reaction (PCR) was carried out to amplify the (GT)_n_ repeat region using a forward primer labeled with a dye (FAM). Forward primer: 5’-FAM-AGAGCCTGCAGCTTCTCAG-3’, reverse primer: 5-AAACAAAGTCTGGCCATAGGAC-3’ (TAG Copenhagen, Denmark). Amplifications were carried out in a total volume of 15 *μ*l containing 1x PCR buffer (50 mM KCl, 20 mM Tris pH 8.4, 2.0 mM MgCl_2_, 0.2 mM cresol red, 12% sucrose, 0.005% (wt/v) BSA (Boehringer-Mannheim, Germany), 5 pmol of each primer, 40 mM dNTPs (Pharmacia LKB, Sweden) and 1.25 U of AmpliTaq polymerase (Perkin Elmer Cetus Corporation, Emeryville, CA, USA). To minimize non-specific amplification, we carried out “touch down” PCR where annealing temperature is higher than target optimum in early PCR cycles followed by a decrease by 0.5°C in every following cycle. Genotyping was performed by analyses of PCR products from amplification of the (GT)_n_ repeat region in a 3500 Genetic Analyzer (Applied Biosystems). 1 μL of PCR product was added to 20 μL formamide (95%) and 0.3 μL GeneScan^™^—600 LIZ^®^ SIZE standard (Applied Biosystems). Fragment length examination was performed using the GeneMapper software version 4.1 (Applied Biosystems).

### Statistical analyses

For our statistical analyses differences in distribution of number of repeats between patients and donors were tested using a Kolomogorov-Smirnov test. Comparisons of groups of patients and discrete variables were done using Fisher’s Exact test and comparisons of groups of patients and continuous variables were performed using the Mann-Whitney test. Overall survival (OS) and progression-free survival (PFS) was estimated by the Kaplan-Meier method. Cumulative incidences of relapse/progression, relapse related mortality (RRM), acute and chronic GVHD were estimated by standard methods for competing risks [10]. A Cox proportional hazard regression model was used for calculating p-values and hazard ratios. Significant associations between genotype and outcome were further investigated in multivariable Cox regression models, restricted to include patient age, sex, donor type (related versus unrelated) and relapse risk (i.e Kahl score for NMA cohort [11] and EBMT for MA cohort [12]) to calculate adjusted hazard ratios and p-values. This was not done on subset analyses on subsets of data due to the low number of cases. In cases of very few events a permutation test was used for calculating differences. All tests were two-sided and p-values below 0.05 were considered significant. IBM SPSS Statistics version 19.0.0 (2010, IBM, Somer, NY, USA) or R Statistical Software version 3.2.2 (2015, R Foundation, Vienna, Austria) were used for all calculations. R package “crr” was used for cumulative incidence calculations and function “permlogrank” in the “clinfunc” package was used for permutation tests.

## Results and Discussion

First, we used fragment analyses to determine the number of (GT)_n_ repeats among 339 recipients and 360 donors as well as 74 healthy donors. Alleles were classified as either short with ≤ 26 (GT)_n_ repeats (S) or long with >26 (GT)_n_ repeats (L) [13]. The (GT)_n_ repeat polymorphism adhered to the Hardy-Weinberg equilibrium in all 3 cohorts (p = 0.55, p = 0.56, p = 0.75). The number of (GT)_n_ repeats ranged from 21 to 39 for recipients and 19 to 38 for donors (S1 Fig). In agreement with previous reports distribution of the alleles was bimodal with the most common alleles consisting of 23 and 30 (GT)_n_ repeats [13]. The distribution of alleles was similar between recipients and donors (p = 0.8) and did not differ from that of healthy donors (p = 0.9 and 0.4, respectively).

**Fig 1:**
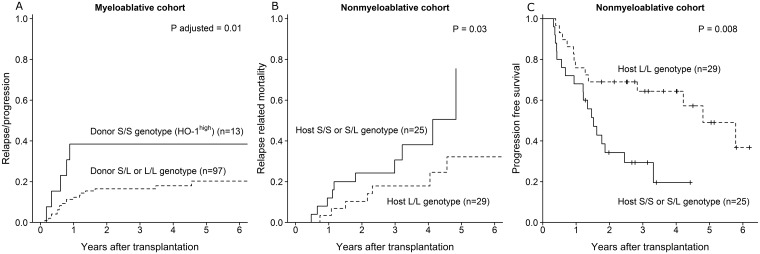
Transplantation outcome. **(A)** Proportion of patients progressing after receiving myeloablative conditioning and grafts from donors with either S/S (solid line) or S/L or L/L (dotted line) genotype. The p value was adjusted for patient sex, age, relapse risk and donor type. **(B)** Incidence of relapse related mortality and **(C)** progression-free survival according to patient HO1 genotype receiving nonmyeloablative conditioning and grafts from an HO-1^low^ genotype donor. S/S + S/L (solid line), L/L (dotted line).

Next, we investigated the association between HO-1 genotype in recipients and donors and clinical outcome after allogeneic MA-HCT. Recipients of grafts from donors with genotype S/S (HO-1^high^) had a significantly higher relapse/progression incidence (time to progression) at 3 years (38%, n = 13) than patients receiving grafts from donors with genotypes S/L or L/L (16%, n = 97) (adjusted hazard ratio (HR) 3.4 (95% confidence interval (CI) 1.3–9.1), p = 0.01, Fig 1A, Table 2), which translated into lower PFS (S/S, 39%; S/L or L/L, 54%; adjusted HR 2.2 (95% CI 1.0–4.46), p = 0.04) and a trend towards increased RRM (S/S, 38%; S/L or L/L, 16%; adjusted HR 2.8 (95% CI 1.0–7.9), p = 0.05) at 3 years (Table 2).

**Table 2:** Uni- and multi-variate analyses of the correlation between donor genotype and transplantation outcome.

	**Myeloablative conditioning (n = 110)**				
	**Unadjusted analysis**			**Adjusted analysis**	
	***At 3 years***^a)^		P^b)^	HR (95% CI)	P
	*Short GT (SS) (donor)*	*Long GT (SL/LL) (donor)*			
Overall survival	38.5% (19.3%; 76.5%)	55.3% (46.1%; 66.2%)	0.34	1.8 (0.8; 3.9)	0.13
Progression-free survival	38.5% (19.3%; 76.5%)	54.3% (45.2%; 65.3%)	0.24	2.2 (1.0; 4.6)	0.040
Time to progression	38.5% (5.4%; 60.0%)	16.5% (8.8%; 23.6%)	0.032	3.4 (1.3; 9.1)	0.013
Relapse-related mortality	38.5% (5.4%; 60.0%)	15.6% (8.0%; 22.5%)	0.049	2.8 (1.0; 7.9)	0.048
Treatment-related mortality	23.1% (0%; 42.9%)	29.2% (19.4%; 37.7%)	0.52	1.2 (0.3; 4.0)	0.81
	***At 1 year***				
Acute GVHD (grade III+)	7.7% (0%; 21.1%)	17.6% (9.6%; 24.9%)	0.26	-	-
Acute GVHD (grade II+)	53.8% (17.0%; 74.3%)	53.9% (43.8%; 62.8%)	0.94	1.0 (0.4; 2.2)	0.99
	**Nonmyeloablative conditioning (n = 250)**				
Overall survival	58.7% (43.3%; 79.4%)	63.6% (57.2%; 70.8%)	0.93	1.0 (0.5; 1.7)	0.90
Progression-free survival	44.5% (23.8%; 70.1%)	56.2% (47.5%; 63.1%)	0.49	1.0 (0.6; 1.9)	0.91
Time to progression	33.8% (9.8%; 51.3%)	25.9% (17.2%; 33.7%)	0.52	1.1 (0.6; 2.5)	0.86
Relapse-related mortality	25.7% (3.3%; 43.9%)	21.0% (12.9%; 28.4%)	0.62	0.9 (0.3; 2.6)	0.89
Treatment-related mortality	21.7% (0%; 38.9%)	17.9% (10.4%; 24.8%)	0.92	1.2 (0.4; 3.5)	0.39
	***At 1 year***				
Acute GVHD (grade III+)	5.7% (0%; 13.1%)	9.0% (5.0%; 12.8%)	0.61	-	-
Acute GVHD (grade II+)	42.9% (23.9%; 57.1%)	30.7% (24.2%; 36.6%)	0.15	1.6 (0.9; 2.9)	0.089

^a)^ For overall survival and progression-free survival, proportion alive Kaplan-Meier estimate. For Time to progression, Relapse-related mortality and Treatment-related mortality cumulative incidence with competing risks. For acute GVHD cumulative incidence with Kaplan-Meier estimate.

^b)^ Log-rank test when Kaplan-Meier estimate used.

No significant difference was observed for OS (S/S, 39%; S/L or L/L, 55%, p = 0.13), and treatment related mortality (TRM) (S/S, 23%; S/L or L/L, 29%, p = 0.81) at 3 years. We did not find a significant association to the incidence of acute GVHD (grade II or higher) (S/S, 54%; S/L or L/L, 54%, p = 0.99) at 1 year or severe acute GVHD (grade III or higher) (S/S, 8%; S/L or L/L, 18%, p = 0.26) at 1 year (Table 2).

In the NMA setting no association between outcome and genotype where observed, when statistical analyses were isolated to host genotype only (Table 2). However, HO-1^low^ genotype (L/L) recipients of HO-1^low^ donors experienced significantly lower RRM (L/L donor into L/L recipient, 18%, n = 29; L/L donor into S/L or S/S recipient, 31%, n = 25; HR 0.3 (95% CI 0.1–0.9), p = 0.03, Fig 1B) and increased PFS (L/L donor into L/L recipient, 64%, n = 29; L/L donor into S/L or S/S recipient, 29%, n = 25; HR 0.4 (95% CI 0.2–0.8), p = 0.008, Fig 1C) at 3 years. Furthermore, we observed a trend towards higher incidence of grade III-IV acute GVHD (L/L, 14%, n = 50, S/L or S/S, 2%, n = 51; p = 0.02, permutation test) at 1 year (data not shown). No other associations between recipient or donor genotype and outcome measures were observed.

We did not observe the same effect of HO-1 genotype in the two cohorts. However, the results were complementary and in support of previously published animal data indicating that HO-1 expression favors induction of regulatory T cells (Tregs) and inhibits proliferation of effector T cells [14], thus mitigating GVT effects. In the MA cohort donorswith an anti-inflammatory phenotype(HO-1^high^ (S/S) genotype) were associated with increased relapse related outcome measures, implying that high levels of the anti-inflammatory HO-1 may suppress GVT effects. In the NMA cohort transplantation using donors with the pro-inflammatory HO-1^low^ (L/L) genotype in equally phenotypically pro-inflammatory HO-1^low^ (L/L) recipients was associated with less relapse and superior PFS, implying augmented GVT effects with less anti-inflammatory drive. The reason for only being able to observe significant association between complementary endpoints, and not the same endpoints, across the two cohorts, may be due to the relatively small cohort sizes and heterogeneity. Due to the same reason the current observations could not be reproduced in joint analyses of the cohorts.

Only 1 study has previously been published investigating the role of HO-1’s genetic variation after allogeneic HCT. In a cohort of 92 patients transplanted with in vivo T-cell depleted grafts from matched related donors MA-HCT, Gerbitz et al. observed that donors with high HO-1 expression (based on (GT)_n_ repeat genotype) and thus increased anti-inflammatory drive, were associated with inferior survival due to grade III-IV acute GVHD≥3 [15]. Gerbitz et al. hypothesize that expression of HO-1 above a certain threshold may lead to an excessive oxidative burst that induces tissue damage [16]. In contrast, our study implied that high expression of HO-1 suppresses the GVT effects.

The apparently inconsistent results observed in the current study and often across genetic association studies may be due to several factors that make direct comparisons difficult. The generally small cohorts in transplant studies increase the risk of type I errors as the effect of single genetic variants usually is modest. Furthermore heterogeneity between patient populations, with differences in treatment regimens, diagnoses, racial admixture and other yet unknown risk factors, as well as use of different laboratory methods and analyses platforms all contribute to an unclear picture.

In conclusion, data from the current study suggest that donor HO-1 genotype may be associated with the GVT effect in both the MA and NMA setting, hereby implying a role for HO-1 in alloreactivity. To validate the significance of these findings further experimental and clinical studies are needed to explain the role of HO-1 in alloreactivity and assess the impact on outcome in a prospective manner with consequences in a clinical setting.

## Supporting Information

S1 Fig Frequency of number of HO-1 (GT)_n_ repeats. We determined the number of (GT)_n_ repeats among 74 normal donors (normal cohort), 360 donors (donor cohort) and 339 recipients (recipient cohort). As every individual carries two alleles we analysed 148 normal donor, 720 donor and 678 recipient alleles.
(TIF)
