# Supplementary figures and images for: (GT)n Repeat Polymorphism in Heme Oxygenase-1 (HO-1) Correlates with Clinical Outcome after Myeloablative or Nonmyeloablative Allogeneic Hematopoietic Cell Transplantation
Source: PLoS One. 2016 Dec 20;11(12):e0168210. doi: 10.1371/journal.pone.0168210 (PMC5172582; doi:10.1371/journal.pone.0168210)

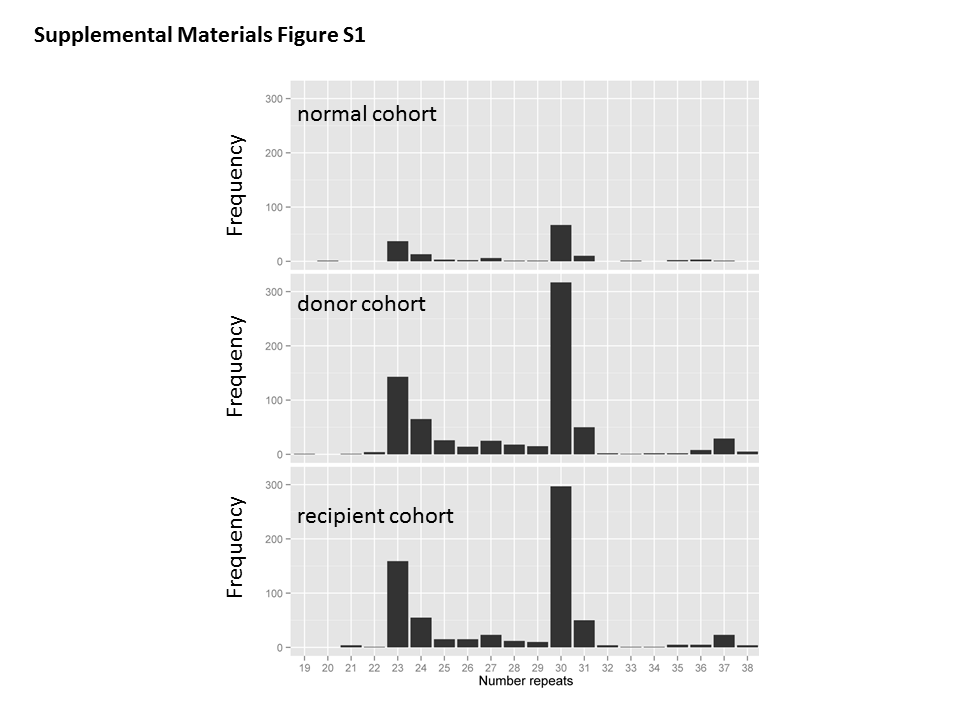

Supplement: S1 Fig — We determined the number of (GT)n repeats among 74 normal donors (normal cohort), 360 donors (donor cohort) and 339 recipients (recipient cohort). As every individual carries two alleles we analysed 148 normal donor, 720 donor and 678 recipient alleles. (TIF) [file pone.0168210.s001.tif]
